# A Wearable Monitor to Detect Tripping During Daily Life in Children with Intoeing Gait

**DOI:** 10.3390/s25206437

**Published:** 2025-10-17

**Authors:** Warren Smith, Zahra Najafi, Anita Bagley

**Affiliations:** 1 Department of Electrical and Electronic Engineering, California State University, Sacramento, CA 95819, USA; zahra.najafi@csus.edu; 2 Shriners Children’s Northern California, Sacramento, CA 95817, USA; abagley@shrinenet.org

**Keywords:** sensors, intoeing gait, tripping, wearable monitor, radio frequency identification (RFID), near-field communication (NFC)

## Abstract

**Highlights:**

**What are the main findings?**

- A miniature, wearable monitor is developed to log tripping and steps taken in intoeing children during two weeks of daily life.

**What is the implication of the main finding?**

- Monitoring tripping in children during daily life will aid clinical assessment and treatment evaluation and improve artificial intelligence understanding of gait.

**Abstract:**

Children with intoeing gait are at increased risk of tripping and consequent injury, reduced mobility, and psychological issues. Quantification of tripping is needed outside the gait lab during daily life for improved clinical assessment and treatment evaluation and to enrich the database for artificial intelligence (AI) learning. This paper presents the development of a low-cost, wearable tripping monitor to log a child’s Tripping Hazard Events (THEs) and steps taken during two weeks of everyday activity. A combination of sensors results in a high probability of THE detection, even during rapid gait, while guarding against false positives and minimizing power and therefore monitor size. A THE is logged when the feet come closer than a predefined threshold during the intoeing foot swing phase. Foot proximity is determined by a Radio Frequency Identification (RFID) reader in “sniffer” mode on the intoeing foot and a target of passive Near-Field Communication (NFC) tags on the contralateral foot. A Force Sensitive Resistor (FSR) in the intoeing shoe sets a time window for sniffing during gait and enables step counting. Data are stored in 15 min epochs. Laboratory testing and an IRB-approved human participant study validated system performance and identified the need for improved mechanical robustness, prompting a redesign of the monitor. A custom Python (version 3.10.13)-based Graphical User Interface (GUI) lets clinicians initiate recording sessions and view time records of THEs and steps. The monitor’s flexible design supports broader applications to real-world activity detection.

## 1. Introduction

### 1.1. Overview

Tripping during walking is a common complaint for children with orthopedic bony malalignment of the lower limbs and for children with cerebral palsy [1,2] and increases the chance of injury, reduced mobility, and psychological problems [2,3]. Tripping can occur when a foot is turned inward (intoed) enough to “cross over” the midline as it swings forward and contacts the contralateral foot. There are a variety of treatments to address tripping during gait, from conservative management with orthoses to surgical bone realignment [1,2]. The efficacy of these treatments needs to be documented. Gait and gait difficulties have been studied in laboratory settings using multicamera motion analysis systems, force plates and pads, and treadmills [4,5,6]. However, children behave differently in a laboratory setting than during daily living [7], sometimes referred to as the “Hawthorne Effect” [8]. To better plan and assess treatment for gait difficulties, clinicians need to know the child’s tripping behavior during daily living. At present, clinicians make do with limited information such as questionnaires completed by the child’s family [9]. To provide better information, clinicians at Shriners Children’s Northern California (SCNC) want quantitative monitoring of tripping in children with intoeing during daily life. This paper describes the development of a wearable monitor to log intoeing-caused tripping, or near-tripping, events by a child during two weeks of everyday living.

The SCNC clinicians want a tripping monitor that takes into account the following considerations: Define a Tripping Hazard Event (THE) to occur when the intoeing swing foot strikes, or comes too close to, the stance foot during gait, meaning that the distance between the swing and stance foot becomes less than a threshold, *D_Th_*. To capture both weekday and weekend activities, continuously record THEs for two weeks of daily living. The monitor also should log steps to provide a context for the THEs. For example, a low THE count might occur because the child is inactive out of fear of tripping and falling. Capture THEs and steps so that the results can be viewed clearly in 24 h plots. The child or parents should not be required to turn the monitor on/off or change/charge its battery. The monitor should be robust enough to endure a child’s activity, should not interfere with that activity, and should be as acceptable in appearance to the child as possible. Based on analysis of video recordings made at the SCNC Motion Analysis Lab, capture THEs between a child’s intoeing foot in swing phase and the lower medial side of the stance foot. Also, capture THEs for swing foot speeds passing the stance foot as high as 5.2 m/s, which can occur during fast walking.

### 1.2. Previous Work

To the authors’ knowledge, no wearable monitor of intoeing-caused tripping during daily life has been developed previously. A number of wearable systems for gait monitoring and analysis have been developed [10,11], such as those using triaxial accelerometers to log a child’s falls and activity level [12,13] and those incorporating the fusion of multiple sensors, such as inertial measurement units (IMUs), magnetometers, pressure sensors, and cameras for monitoring movement behavior [14,15] and measuring gait variables including cadence, stride length, walking speed, and gait failure [16,17,18]. Some systems have addressed the problem of tripping due to inadequate toe ground clearance by incorporating Time-of-Flight (ToF) distance sensors [19,20].

Some wearable systems measuring inter-foot distance have been developed. In one multi-sensor system, medially aimed ToF distance sensors were strapped on the legs above the ankles to detect leg movements [16]. A shoe-mounted ToF sensor was used to measure inter-foot distance to assess gait stability [21]. This ToF sensor (Model GP2Y0A41SK0F, Sharp Corp., Osaka, Japan) has a distance range of 40 mm to 300 mm and a maximum sampling rate of 60 Hz [22]. Another device with ToF sensors mounted medially on shoes was developed to measure step count [23]. This ToF sensor (Model VL6180X, STMicroelectronics, Geneva, Switzerland) has a distance range of 10 mm to 100 mm and a maximum sampling rate of 50 Hz [24]. The device also has been used in a multi-sensor system to estimate stride width to determine a subject’s base of support [25].

### 1.3. RFID/NFC Measurement of Inter-Foot Distance

**Figure 1:**
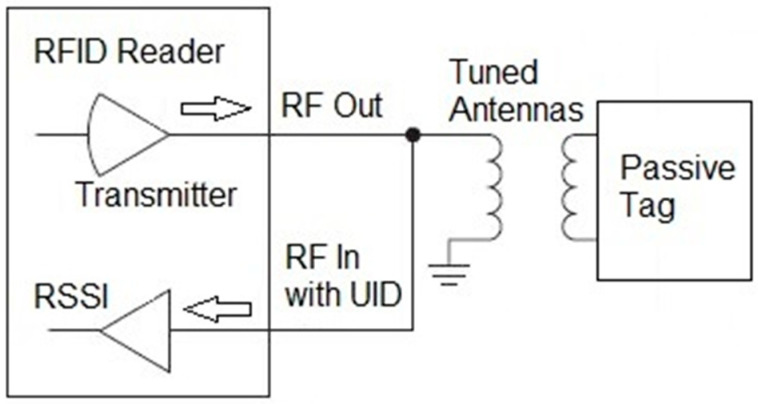
A common RFID arrangement.

Radio Frequency Identification (RFID)/Near-Field Communication (NFC) technology [26,27,28] is promising to measure foot-to-foot distance. This technology is well-developed and proven, widely used, readily commercially available, and low-cost. The RFID readers are small, and the tags are small, thin, lightweight, and of many sizes and shapes. Low levels of RF are involved, comparable to those for credit card or merchandise tag readers. In a common arrangement, shown in Figure 1, an RFID reader transmits a radio frequency (RF) pulse to a passive tag. If the reader is close enough to the tag, the RF powers the tag, which sends back its unique identification number (UID). The reader also can use its internal Received Signal Strength Indicator (RSSI) to determine the amplitude of the return signal from the tag.

The concept for a tripping monitor was to mount a battery-powered RFID reader with its antenna on the shoe of a child’s intoeing foot and a passive tag target on the contralateral shoe. The TRF7970A 13.56 MHz RFID/NFC transceiver integrated circuit (Texas Instruments, Dallas, TX, USA) was chosen as the reader, using the ISO/IEC 15693 protocol [29]. The plan was to utilize a tag’s UID for positive confirmation that the reader was interacting with the shoe tag. The specificity of this reader-tag interaction is important, because the tripping monitor will be worn by a child in uncontrolled home and community environments. The RSSI then would measure the amplitude of the return signal, which increases with decreasing distance between the reader antenna and the tag. A return signal amplitude threshold would be set to correspond to the desired distance threshold, *D_Th_*. If this amplitude were exceeded, a THE would be detected.

To determine whether a satisfactory tripping monitor could be built using the arrangement shown in Figure 1, an RFID reader consisting of a TRF7970A BoosterPack (Texas Instruments, Dallas, TX, USA) plugged onto the MSP-EXP430G2ET LaunchPad kit (Texas Instruments, Dallas, TX, USA) and a Tag-HF-1 D03 (Texas Instruments, Dallas, TX, USA), ISO15693 tag having a 33 mm-diameter circular antenna were used. Firmware provided by Texas Instruments was downloaded into the kit’s MSP430G2553 (Texas Instruments, Dallas, TX, USA) microcontroller (MCU) to operate the reader. The reader has a rectangular 43 mm × 24 mm antenna on the BoosterPack printed circuit board. The areas of the reader and tag antennas were considered to approximate what would be used on a child’s shoes. When the reader antenna and tag were positioned parallel to each other and spaced 10 mm apart, the reader could detect the tag over a lateral range of 45 mm. At the foot-passing speed of 5.2 m/s, the tag would be in a 45 mm lateral detection range for 8.6 ms. With the ISO/IEC 15693 protocol, including Start of Frame and End of Frame durations, it takes the reader 6.76 ms to request a tag’s UID, and, in the high-bit rate mode, it takes the tag 5.14 ms to return its UID, for a total transmit/receive time of *T_tr_* = 11.9 ms [29]. This time is longer than the 8.6 ms available for detection, even assuming the best case that reader transmission begins just as the reader antenna enters the 45 mm range. To ensure tag detection, the reader should be able to sample multiple times during the 8.6 ms available. A reader sample rate of 300 sniffs/s meets this requirement, because that would allow the reader to sample at least twice during the available time window. Therefore, it was necessary to abandon an RFID system that depends on obtaining a positive identification of a tag’s UID.

An alternative approach to using the RFID reader was considered. A low-power method using RFID/NFC for proximity detection has been developed that is preferable to using a mechanical or optical switch or a magnetic or capacitive sensor [30,31]. The method is commonly used by an initiator, such as a mobile phone, to detect the presence of a passive target, such as a payment card. The initiator emits a short burst of NFC-frequency RF, then measures the decay time of the envelope-detected RF. A change in the decay time is taken to indicate the proximity of a card.

The phenomenon underlying this proximity detection was utilized to implement a tripping monitor. Because the reader and tag antennas are tuned to the same frequency, the reader antenna RF amplitude increases in a continuous fashion as it approaches the tag. This amplitude increase was used to measure reader-tag distance. Because the reader no longer reads data from the tag, it is more appropriately called a “sniffer.” The tuned nature of the reader and tag antennas offers a substitute to the UID for positive confirmation of the tag’s presence, as desired for reliable operation in uncontrolled daily living environments.

### 1.4. Step Counting

To enable the monitor to count steps, the plan was to use a Force-Sensitive Resistor (FSR) method developed and validated in previous projects [32,33]. An FSR 402 (Interlink Electronics, Fremont, CA, USA) is inserted beneath the shoe insole of the intoeing foot. The FSR is in a voltage-divider circuit supplied by 3.3 V with output voltage *V_FSR_* that decreases with increasing foot pressure. When the intoeing foot lifts off the ground, *V_FSR_* rises from below a low threshold of 0.5 V to above a high threshold of 1.5 V. A step is counted when the foot is set down and *V_FSR_* again drops below the low threshold. Clinical experience may suggest changes in these thresholds.

### 1.5. RFID Antenna

**Figure 2:**
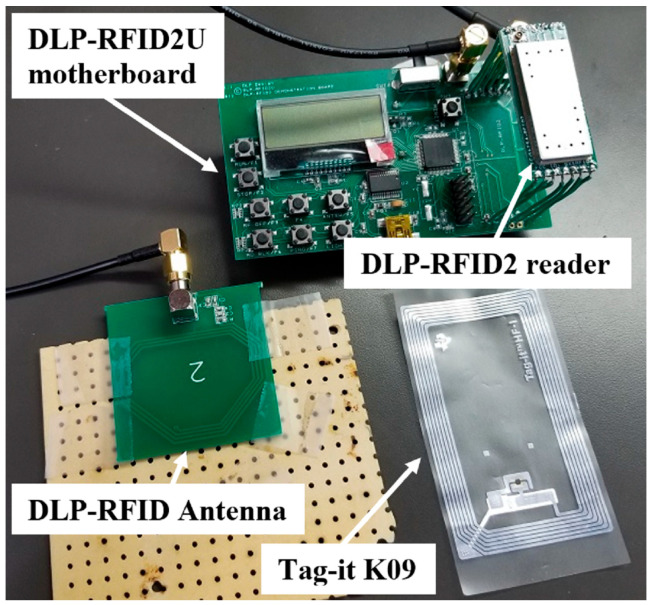
DLP-RFID2-EDK evaluation kit reader used to test candidate sniffer antennas.

**Figure 3:**
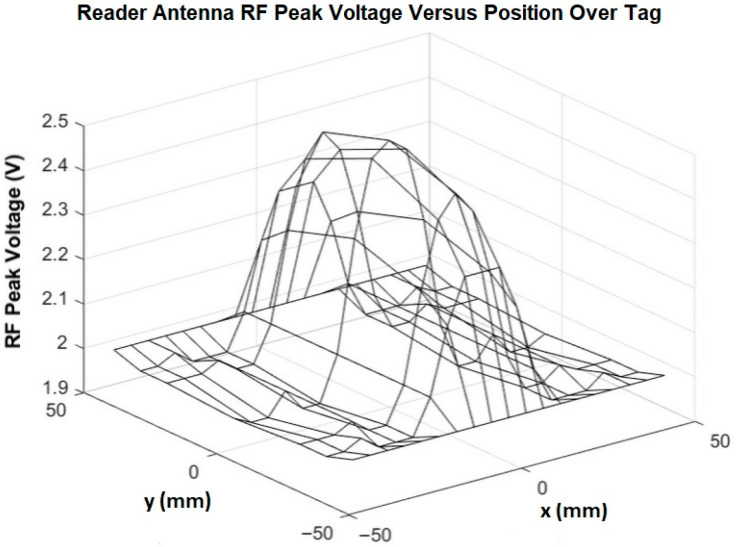
A plot of antenna RF peak voltage when a reader antenna was spaced 2 mm above the tag in Figure 2 and moved laterally (*x*-axis) and longitudinally (*y*-axis) over it.

The DLP-RFID2-EDK evaluation kit (DLP Design, Inc., McKinney, TX, USA), with its 50 Ω external antenna port, was used to test candidate sniffer antennas. The kit consists of a DLP-RFID2U motherboard, a DLP-RFID2 reader for mounting on the motherboard, and a variety of printed-circuit-board external antennas. Figure 2 shows the DLP-RFID2U (upper) with one of its external antennas (lower left) next to a 70 mm × 45 mm Tag-it K09 (Texas Instruments, Dallas, TX, USA) (lower right). Figure 3 shows a plot of antenna RF peak voltage when the antenna was spaced 2 mm above the tag and moved laterally (*x*-axis) and longitudinally (*y*-axis) over it. After testing a variety of antennas, the thin, flexible, 53 mm × 37 mm FXR.01.A antenna (Taoglas, Ltd., Dublin, Ireland) was identified as a promising candidate. It can conform to the shoe, and it has a built-in impedance-matching circuit for a 50 Ω port.

### 1.6. Tag Target

**Figure 4:**
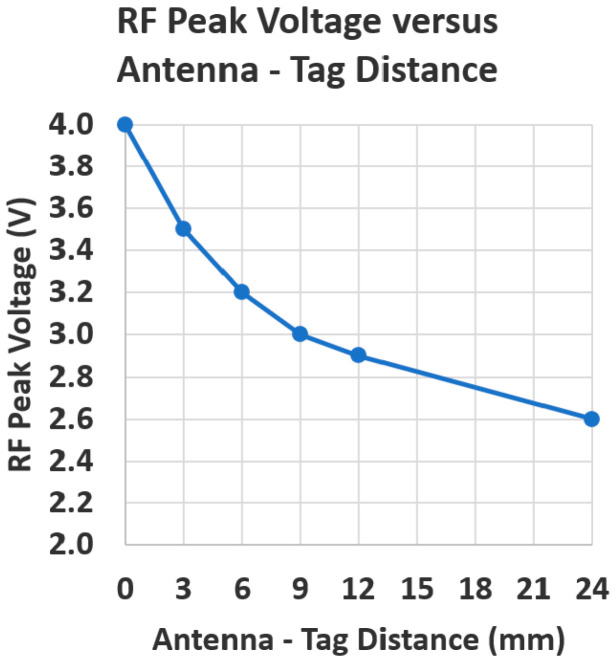
Antenna RF peak voltage versus distance between an FXR.01.A external antenna on the DLP-RFID2-EDK and an NTAG215 NFC tag.

With the sniffer, an NFC tag can be used, such as the 50 mm × 30 mm × 0.8 mm NTAG215 NFC tag (TimesKey NFC, Guangdong, China). Figure 4 shows the RF peak voltage versus distance between an FXR.01.A external antenna on the DLP-RFID2-EDK and a coaxial, parallel NTAG215 NFC tag.

**Figure 5:**
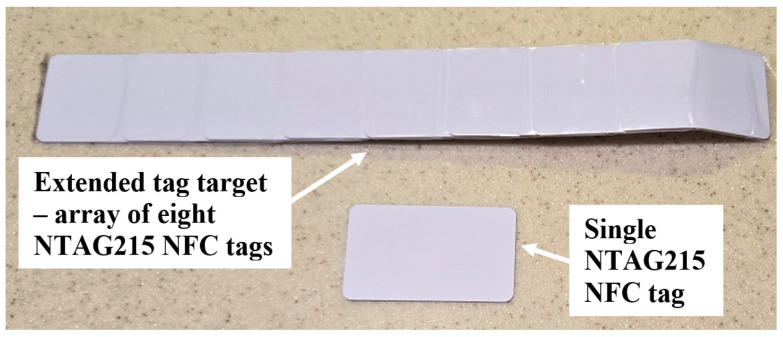
A single NTAG215 NFC tag (bottom) and an extended tag target (top) made from eight of these tags.

**Figure 6:**
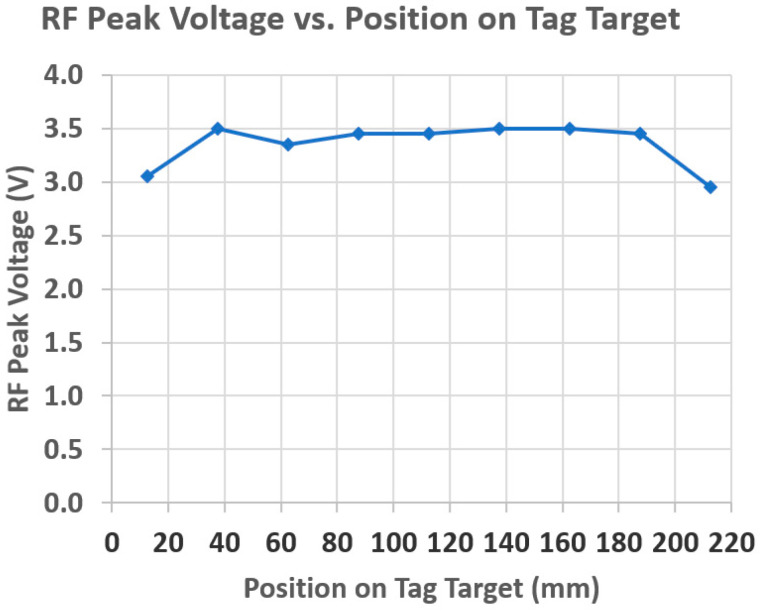
FXR.01.A antenna RF peak voltage versus position, from left to right, along the extended tag target shown in Figure 5 for a 6 mm antenna–tag target separation.

It is not known just where the intoeing swing foot will strike or most closely approach the stance foot. It was found that the NFC tags can be combined to create a larger tag target to increase the likelihood of detection. Given the SCNC guidelines regarding where the intoeing foot is likely to strike the stance foot, an extended tag target was prepared for placement along the lower medial side of the non-intoeing foot. Figure 5 shows a single NTAG215 NFC tag (bottom) and an extended tag target consisting of eight of these tags (top). The rightmost tag in the extended tag target was curved to conform to the heel of the shoe. Overlapping the 50 mm-long tags by 25 mm produces the most uniform effect on the antenna RF amplitude along the tag target, as measured by using an external FXR.01.A antenna with the DLP-RFID2-EDK kit. Figure 6 shows RF peak voltage along the tag target in Figure 5, from left to right, for a 6 mm separation of the antenna from the tag target. For comparison, for the single tag shown at the bottom in Figure 5, the RF peak voltage was 3.15 V for a 6 mm separation. If desired, the tag target can be extended to be wider, as well as longer.

## 2. Materials and Methods

### 2.1. Prototype Sniffer-Based Tripping Monitor

#### 2.1.1. Block Diagram

**Figure 7:**
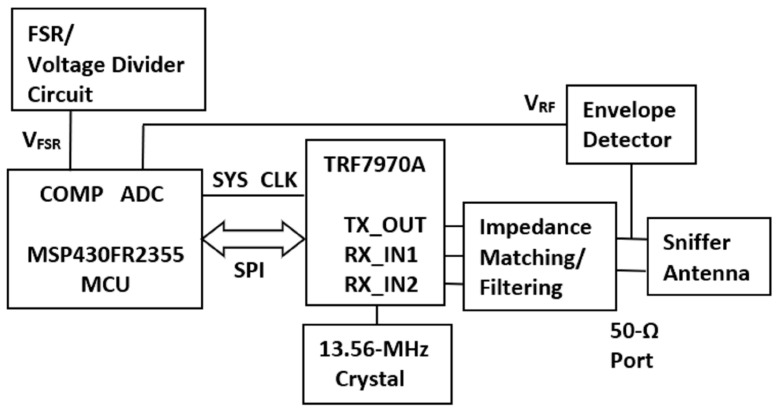
Block diagram of sniffer-based tripping monitor.

The goal was to implement and test the feasibility of an RFID/NFC sniffer-based tripping monitor in preparation for preliminary testing by SCNC clinicians on children with intoeing gait. Figure 7 shows a block diagram of the prototype tripping monitor. It consists of an MSP430FR2355 microprocessor (Texas Instruments, Dallas, TX, USA) in communication and synchrony with a TRF7970A RFID integrated circuit (Texas Instruments, Dallas, TX, USA). A 13.56 MHz crystal (9B-13.560MAAE-B, TXC Corp., Taoyuan City, Taiwan) provides the appropriate RF. An FXR.01.A “sniffer” antenna is connected to the RFID integrated circuit via custom impedance-matching circuitry. A custom envelope detector circuit finds the antenna RF peak voltage and feeds it to the analog-to-digital converter (ADC) of the MCU. For step detection, FSR circuitry as described in Section 1.4 feeds output voltage *V_FSR_* to the comparator (COMP) of the MCU.

#### 2.1.2. Sensor

The core sensing concept of the monitor is that the amplitude of the sniffer antenna’s RF voltage increases as the distance between the sniffer antenna and an NFC tag tuned to the same frequency decreases. Therefore, this increase in sniffer antenna voltage can be used to measure sniffer antenna-to-tag distance.

#### 2.1.3. Data Processing and Storage

Starting at rest, when the intoeing foot lifts off the ground, the increase in *V_FSR_* triggers the MCU to initiate a time window for sniffing. During this window, the MCU commands the RFID integrated circuit to emit 43 μs pulses of 13.56 MHz RF at the rate of 300 sniffs/s. This sniffing rate is to ensure that THEs are detected during fast walking. The envelope-detection circuit finds the RF peak amplitude voltage, *V_RF_*. At 20 μs after each RF pulse ends, the MCU uses its analog-to-digital converter (ADC) to sample *V_RF_* and compares it with a voltage threshold, *V_Th_*, set to correspond to a desired distance threshold, *D_Th_*. If the RF voltage exceeds *V_Th_*, a THE is detected. The MCU stores the number of THEs in its nonvolatile memory in 15 min epochs. This epoch length is to ensure that plots of 24 h of data are uncluttered. The sniffing window ends if a THE is detected, if the intoeing foot sets back down (as determined by the FSR), or after a preset time interval of 1 s. The MCU also uses *V_FSR_* to detect and count steps taken, which it also stores in the 15 min epochs. Clinical experience may suggest a different epoch length.

### 2.2. Sniffing Rate Testing

**Figure 8:**
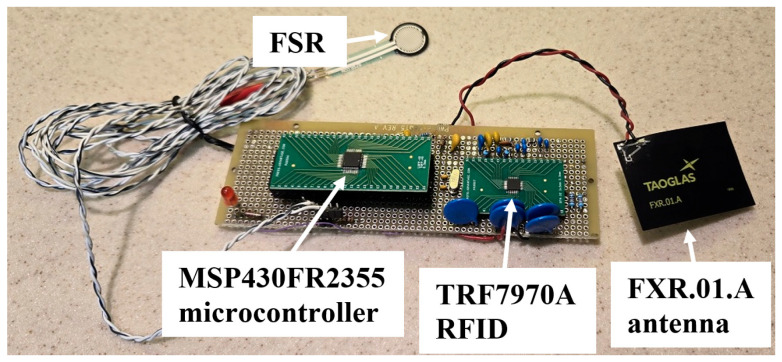
Prototype tripping monitor.

The breadboard tripping monitor shown in Figure 8 was used for laboratory testing. The raised board on the left in the figure holds the MSP430FR2355 MCU, and the raised board on the right holds the TRF7970A RFID integrated circuit. An FXR.01.A antenna is shown on the right, and an FSR is shown above the protoboard.

**Figure 9:**
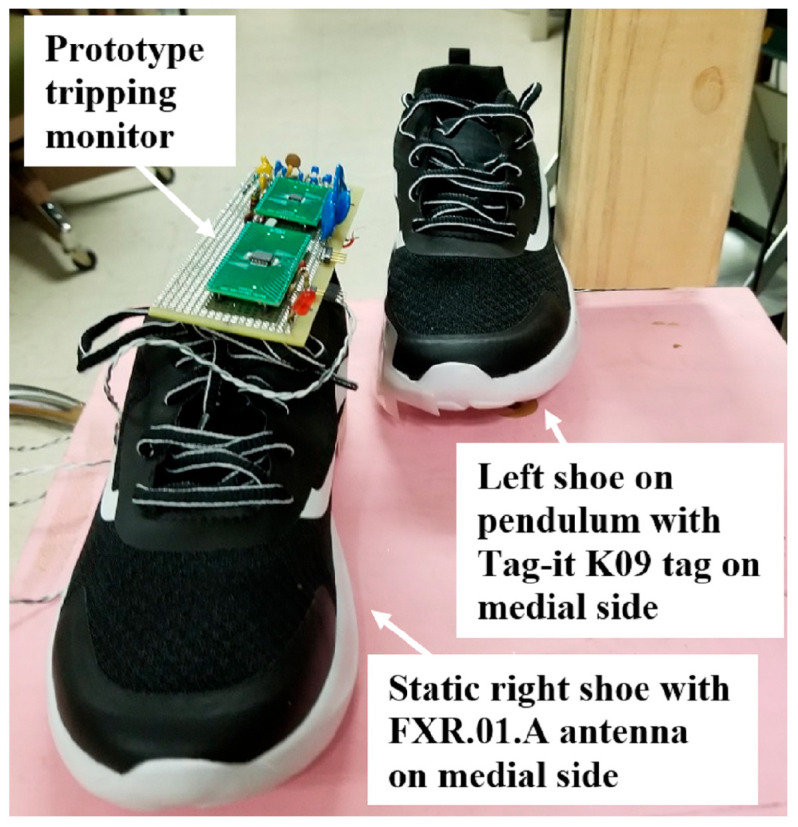
Laboratory simulation of swing and stance feet to test whether the tripping monitor can detect THEs at high swing speeds. The left-foot shoe with tag, mounted on the bottom of a pendulum, swung past the stationary right foot shoe with the tripping monitor.

The tripping monitor was tested to see whether, at 300 sniffs/s, it can detect THEs for the expected speeds at which the intoeing swing foot passes the stance foot. A meter-long wooden 2 in. × 4 in. pendulum was suspended from a hinge, and a left shoe was attached to the bottom of the pendulum to simulate a swing foot. Pendulum equations showed that desired swing foot speeds could be achieved by releasing the pendulum from specified angles relative to downward vertical. For example, a starting angle of 110° corresponds to an expected swing foot speed of 6.28 m/s. The right shoe was fixed on a platform to simulate a stance foot. Figure 9 shows the two shoes. The prototype tripping monitor’s FXR.01.A antenna was mounted on the lower medial side of the right shoe, and a Tag-it K09 was mounted on the lower medial side of the left shoe. The custom MCU software was modified to light the red light-emitting diode (LED) shown on the lower left corner of the protoboard in Figure 8 when a THE was detected.

The pendulum was lifted to a starting angle, and pressure was exerted on the FSR by manually pinching it (hence the long FSR cable shown in Figure 8). Then, the FSR was released to simulate the lifting of the swing foot, and the pendulum was released at the same time. The swing shoe grazed the stance shoe to simulate a THE. The speed of the swing shoe as it grazed the stance shoe was measured using the output voltage of a photodetector sensing the reflection of a light source off a target mounted on the pendulum, and the red LED showed whether a THE was detected.

### 2.3. Battery Capacity Testing

To determine the tripping monitor’s needed battery capacity, the electrical current during monitor operation was found by measuring the voltage across a 1 Ω resistor in series with a 3.3 V supply powering the monitor. Also, to determine how long the monitor could successfully operate on battery power, computer-controlled simulated steps were applied to the FSR of the breadboard tripping monitor powered by a 3.7 V LP102530JU 700-mAh lithium polymer rechargeable cell (Jauch Quartz, Villingen-Schwenningen, Germany).

### 2.4. Step Count Testing

To determine step count accuracy, the number of steps recorded by the tripping monitor during the duration testing described in Section 2.3 was compared with the number of computer-controlled simulated steps applied.

### 2.5. Human Participants Study

**Figure 10:**
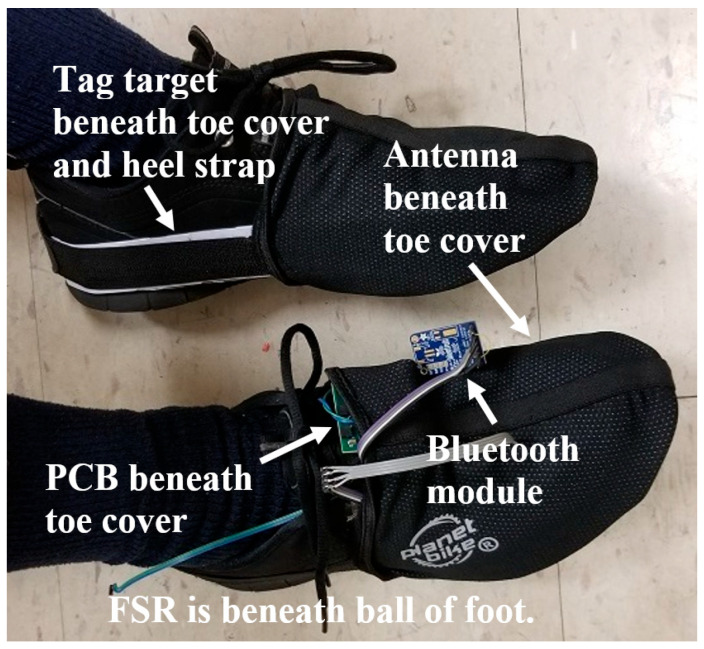
Bicycle toe covers used to attach the tripping monitor to shoes. The right shoe has PCB electronics, sniffer antenna, FSR, and Bluetooth serial communication module. The left shoe has the extended tag target.

A human participant study, IRB-approved and in accordance with the Helsinki Declaration of 1975, as revised in 2024, and to which the participants gave written informed consent, was performed to provide an estimate of the tripping monitor’s accuracy in counting THEs and steps taken and determine how well it could survive being worn on shoes. A printed circuit board (PCB) version of the tripping monitor was created in preparation for attaching the monitor to shoes. The initial goal was to develop an attachment method that did not alter the shoes. To achieve this aim, strap-on bicycle toe covers were tried, such as shown in Figure 10 (Planet Bike, Madison, WI, USA). In the figure, the PCB version of the tripping monitor and its FXR.01.A sniffer antenna is inserted beneath the toe cover of the right shoe, with the PCB on the upper toe surface and the antenna on the anterior medial surface. An FSR is placed in the right shoe at the location of the ball of the foot, with its cable routed out of the shoe for connection to the PCB.

An extended target of NTAG215 NFC tags is inserted beneath the toe cover and its heel strap on the medial side of the left shoe. Also shown on the right shoe is a small Bluetooth module to provide wireless serial communication so that a laptop can issue commands to the tripping monitor and upload monitor data.

Four healthy adult participants tested shoe-mounted tripping monitors. The THE detection distance threshold was set to *D_Th_* = 6 mm. In the monitor MCU, the sniff voltage is converted to an ADC sniff value, *SV*. The monitor’s THE sniff value threshold, *SV_Th_*, corresponding to *D_Th_*, was experimentally determined for each participant. Then, in random order, either a 4 mm spacer (so *D* < *D_Th_*, a “positive” THE detection event) or an 8 mm spacer (so *D* > *D_Th_*, a “negative” event) was affixed over the sniffer antenna. In each case, participants were asked to stand and scuff the sniffer antenna-with-spacer on one shoe against the tag target on the other shoe five times while their feet were video recorded. Participants also were asked to walk normally, then fast, around an oval track. While walking, they were asked to scuff the sniffer antenna-with-spacer against the tag target at marked intervals along the track. Again, their feet were video recorded.

### 2.6. Graphical User Interface (GUI)

A Python (version 3.10.13)-based Graphical User Interface (GUI) for a Windows laptop was developed to enable clinicians to initiate a recording session and to upload, store, and view the resulting THE and step data.

## 3. Results

### 3.1. Sniffing Rate Results

The highest swing speed tried was 6.0 m/s, above the upper limit of 5.2 m/s observed at SCNC in fast-walking children. The tripping monitor sampling at 300 sniffs/s successfully detected simulated THEs at the foot speed of 6.0 m/s. The results are promising, but more testing is needed for confirmation and to investigate even higher sampling rates.

### 3.2. Battery Capacity Results

When the tripping monitor is in its idle mode, the MCU is in low power mode LPM3, with a current of 1.3 μA [34]. When the monitor enters its sniffing mode, current rises to 41 mA during each 43 μs RF pulse, on top of an average current of 3 mA. Assuming that a child walks 10,000 steps/day (5000 for the intoeing foot), that the intoeing foot swing phase duration is 0.3 s, and that the monitor sniffs at 300 sniffs/s during the swing phase, then the required battery capacity is 20 mAh. When the FSR is pressed, a current of 0.3 mA flows through its circuit. If a child stands for 5 hr/day, an additional battery capacity of 21 mAh is required, resulting in a total capacity requirement of 41 mAh. This value is far less (by a factor of 17) than the 700 mAh battery capacity.

The laboratory results from applying computer-controlled simulated steps to the FSR of a tripping monitor add further confirmation of adequate battery life. Assuming 10,000 steps/day, the battery-powered prototype tripping monitor successfully operated for over 30 days, well beyond the desired two-week recording capability. Clinical experience will further guide whether battery capacity needs to be increased or can be reduced.

### 3.3. Step Count Results

In the computer-applied step study, the tripping monitor recorded the same number of steps as were applied, thus verifying that the monitor counts steps accurately.

### 3.4. Human Participants Study Results

**Figure 11:**
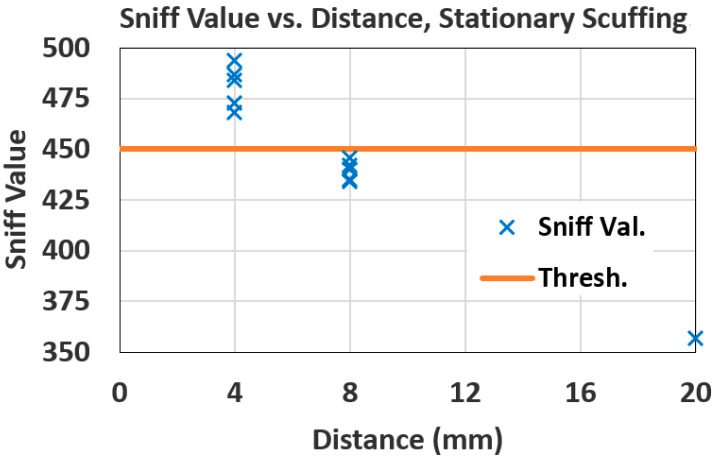
Stationary scuffing results for one adult human participant.

Figure 11 shows a scatterplot of the results for stationary scuffing for one participant. Shown vertically is the MCU ADC sniff value, *SV*. For this participant, the *SV* threshold estimated beforehand for the distance threshold of *D_Th_* = 6 mm was *SV_Th_* = 450. When the sniffer antenna was far away from any tag, *SV* was 357 (shown on the plot at 20 mm). The plot shows that all the scuffs with a 4 mm spacer were correctly detected as THEs (all *SV* values above *SV_Th_* = 450), resulting in a True Positive Percentage (TPP), or sensitivity, of 100%, and the *SV*s for all the scuffs with an 8 mm spacer were correctly below *SV_Th_* = 450, resulting in a True Negative Percentage (TNP), or specificity, of 100%. As statistical evidence that this separation is not chance, an unpaired, one-tailed Student’s *t*-test assuming equal variances gives a t value of 9.56 and *p* < 0.0001 [35]. Perfect separation of positives (4 mm spacer) and negatives (8 mm spacer) occurred for three of the four participants. One participant achieved a TPP of 60% and a TNP of 100%. Examination of the video recordings for this participant showed that the low TPP value occurred because the first two attempted scuffs were off-target.

For the more difficult task of scuffing while walking, the shoe monitor wiring failed during testing for the first participant. The remaining participants achieved an average TPP of 58% and a TNP of 100%. This lower TPP likely occurred because accurate scuffing is difficult while walking. The monitor overestimated the number of steps taken during walking by 6%, suggesting more filtering of the FSR voltage and/or adjustments of the high and low thresholds are needed.

The data in Figure 11 can provide an estimate of the sensitivity, or TPP, for THE detection. As a measure of *SV* scatter for a given *D*, use the average of the standard deviations of sniff values for the given 4 mm and 8 mm spacers in Figure 11. This value is *SD_SV_* = 7.7. For the data in the figure, the *SV* versus *D* regression line is *SV* = −10.45 *D* + 523. Therefore, as a measure of *D* scatter for a given *SV*, the standard deviation for *D* values is *SD_D_* = *SD_SV_*/10.45 = 0.45 mm. For the regression line, a THE distance threshold of *D_Th_* = 6 mm corresponds to a sniff value threshold of *SV_Th_* = 460. Assume for simplicity a uniform distribution of *D* for a given *SV*, resulting in a range [35] of *D* values of 12^½^ × *SD_D_* = 1.56 mm. Also assume a uniform distribution of *D* values from 0 to 6 mm for THEs. Then, given that *D* is less than *D_Th_*, the probability that *SV* is greater than *SV_Th_*, and therefore that a THE is detected, is 0.94, resulting in an estimated sensitivity for THE detection of TPP = 94%.

An estimate of the specificity, or True Negative Percentage (TNP), for THE detection depends on the intra-individual variability of foot-to-foot distances during normal gait. As an example of such variability, for 64 typically developing children, aged 2, 3, and 6 years, average stride width was measured in a laboratory setting to be 80 mm, and intra-individual variability as a coefficient of variation was 25% [5]. Assuming a Gaussian distribution, the probability of a stride width less than 6 mm, that is, of a THE false positive, is 0.0001 [35], resulting in an estimated specificity of THE detection of TNP = 99.99%. Intra-individual variability of foot-to-foot distance in typically developing children may be greater during daily life, and therefore TNP may be lower. Still, the monitor will be useful to compare tripping before and after intoeing treatment, even in the presence of some false positives and false negatives.

### 3.5. GUI Results

**Figure 12:**
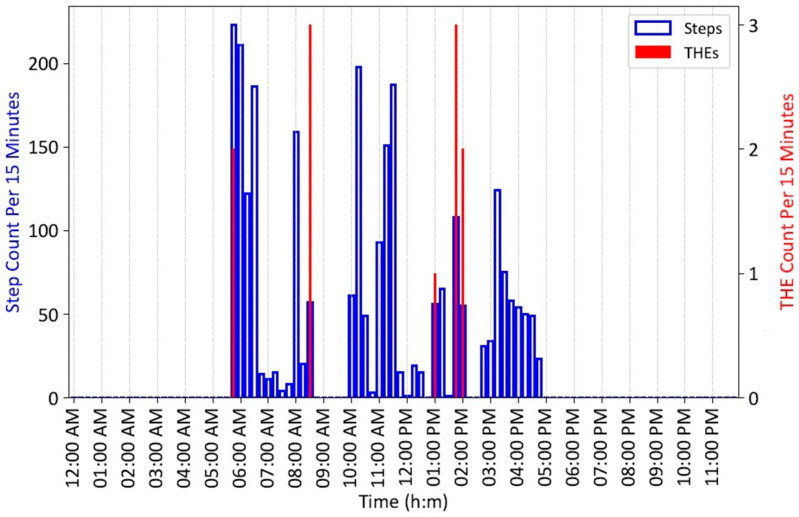
THE and step data for an adult wearing a tripping monitor for a day.

The Python (version 3.10.13)-based GUI developed for a Windows laptop facilitates starting recording sessions and uploading, saving, and displaying the resulting time records of THE and steps data. Figure 12 shows an example recording from the GUI for an able-bodied adult wearing a tripping monitor during a day of everyday living. To generate THE activity, the participant intentionally scuffed the sniffer antenna against the tag target at times during the day. The blue vertical bars show the number of steps taken in each 15 min interval, with counts shown on the left vertical axis, while the red bars show the number of THEs detected, with values indicated on the right vertical axis. The choice of 15 min epochs provides an uncluttered representation of 24 h of activity, and the numbers of steps taken and THEs per epoch can be clearly read. Clinical experience may suggest changing epoch length.

### 3.6. Improved Monitor

**Figure 13:**
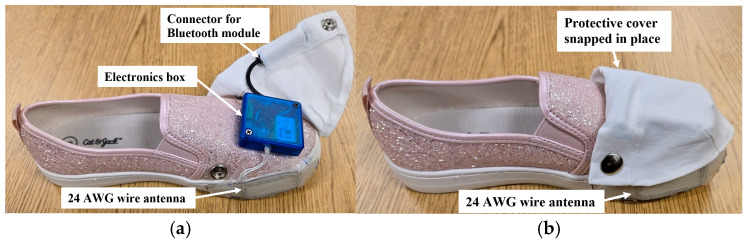
Shoe with tripping monitor. (**a**) Uncovered blue electronics box on upper toe surface of shoe. The thick black cable extends from inside the box to a barrel socket for connecting a Bluetooth module. The FSR cable extends up into the box from a small hole in the lower, lateral side of the shoe. The white antenna wires extend up into the box from the medial side of the shoe. (**b**) Cover snapped in place over the electronics box.

**Figure 14:**
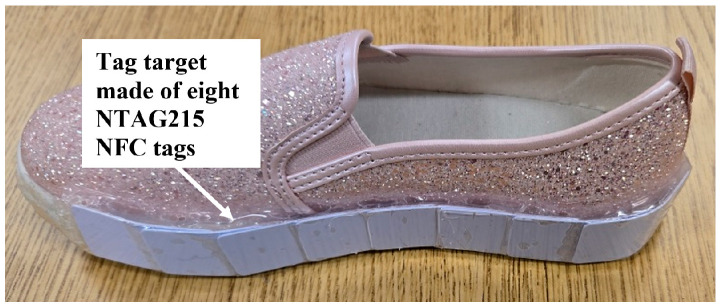
Extended tag target conforming to the curvature of a shoe.

A major lesson learned from the human participants study is that the tripping monitor mounted on shoes is exposed to significant mechanical stress. Therefore, to prepare the tripping monitor for the SCNC clinical study, its durability and its attachment to shoes were improved. A smaller PCB version of the tripping monitor was developed and enclosed with its battery in a plastic electronics box. This box is glued to the upper toe surface of the shoe and protected by a snap-down cloth cover. Figure 13 shows a left shoe with a mounted electronics box, uncovered (a) and covered (b). For protection, all electrical connections are enclosed inside the box. A short cable with a 3.5 mm, four-conductor barrel socket extends from inside the box to connect a separate, self-powered Bluetooth module. This module enables wireless serial communication so that the monitor can receive commands to initiate recording and upload the resulting data. The FSR is protected under the insole of the shoe, and, to keep its cable away from the foot, it passes through a small hole low on the lateral side of the shoe and up to the electronics box. A more rugged sniffer antenna, visible on the lower, forward, medial side of the shoe in the figure, is made from insulated, solid core 24 AWG wire. The antenna, and a 0.4 mm-thick protective plastic cover, are glued to the shoe using Shoe Goo^®^ (Eclectic Products, Eugene, OR). The ends of the antenna wire extend to its impedance-matching circuit inside the plastic box. The tag target also is glued on using Shoe Goo^®^. Figure 14 shows a right shoe with an attached tag target.

**Figure 15:**
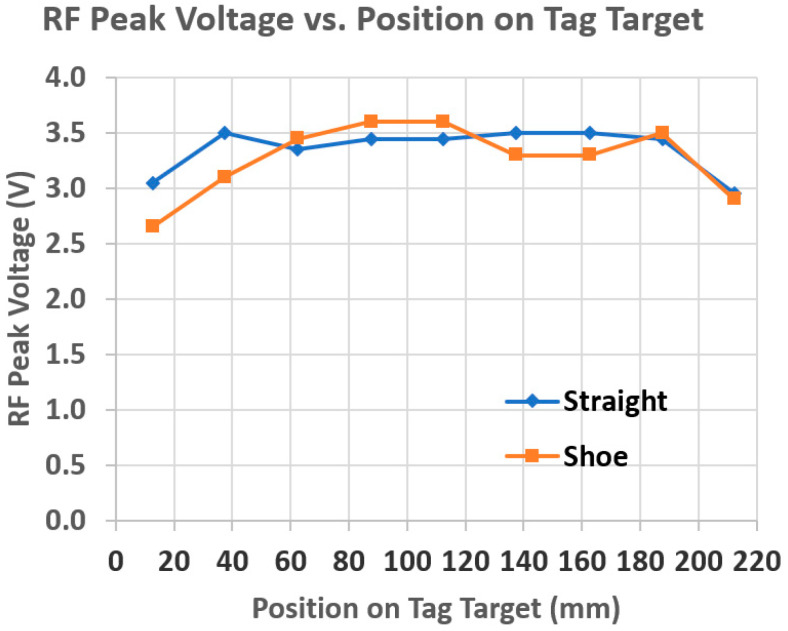
Comparison of FXR.01.A antenna RF peak voltage versus position along a curved extended tag target (labeled “Shoe”) with that for the straight extended tag target shown in Figure 6 (labeled “Straight”) for a 6 mm antenna–tag target separation.

Conforming the extended tag target to the shoe causes variations in the sniff antenna RF peak voltage with position. Figure 15 compares the FXR.01.A antenna RF peak voltage versus position along a curved tag target with that shown in Figure 6 for a straight tag target for a 6 mm antenna-tag target separation. The voltage variations caused by curvature resulted in ±2 mm equivalent deviations from the desired 6 mm value for *D_Th_*. Thus, for example, where the tag target is convex, the antenna would need to be within 4 mm, instead of 6 mm, for THE detection. This smaller distance is still clinically reasonable for THE definition/detection. Though not implemented, tests showed that the voltage variations can be leveled out by placing segments of conductive foil over the tag to reduce the higher voltages.

Monitor size and weight are low. On the intoeing shoe in Figure 13, the mass of the 40 mm × 40 mm × 20 mm box of electronics is 50 g, that of the 50 mm × 25 mm × 1 mm antenna is 2 g, and that of the FSR is 0.25 g. Thus, the monitor increases the mass of the child’s shoe by about 20%. On the contralateral shoe, the mass of the eight-tag 225 mm × 30 mm × 1 mm target is 15 g. Monitor cost is modest. For the intoeing shoe, the parts, including the battery and FSR, cost USD 59.12. On the contralateral shoe, the NFC cards cost USD 0.28 each, so a tag target of eight cards costs USD 2.24. The authors know of no other wearable intoeing-caused tripping monitor for comparison.

## 4. Discussion

The results, though preliminary, are promising. With further confirmation and improvement, this technology can provide a valuable augmentation to observations made in more restrictive laboratory settings and anecdotal information supplied by families.

The monitor is entirely shoe mounted. It uses low-power RF sniffing. Pressure sensing to provide a time gate for sniffing further reduces power demand and the probability of false detection. The tuned nature of the reader and tag antennas ensures the tag detection specificity required for the monitor’s successful operation in an uncontrolled everyday world. The tag target size and shape can be adapted to the clinical need. The monitor’s log of steps taken provides clinicians context for interpreting THE count. A dedicated laptop GUI assists clinicians with initiating recordings, uploading data, and reviewing visualizations of the time course of THEs and steps taken. The tripping monitor is now undergoing preliminary testing at SCNC, collecting data during two weeks of daily living in children with intoeing.

The monitor electronics can be further miniaturized and housed in an ergonomic custom enclosure, and the tag target can be made of thinner, more flexible custom tags. The parameters of the monitor, such as distance threshold, *D_Th_*, sniffing rate, time window for sniffing, voltage thresholds for step counting, and epoch duration for accumulating THEs and steps, can be adjusted as guided by clinical experience.

Multi-sensor fusion systems that incorporate the tripping monitor can provide context, such as the subject’s activity, to aid in interpreting the tripping data. In return, the availability of the tripping monitor’s real-world data for AI learning can improve its algorithms for gait interpretation, enhance predictive modeling of fall risk, help in automatic classification of gait abnormalities, support individualized treatment recommendations, and contribute to the development of adaptive assistive technologies that respond to real-time movement patterns. The monitor’s technology can be integrated into patients’ rehabilitation protocols to provide real-time encouragement and feedback on progress achieved, such as by means of sounds or lights, to remind the children to adjust their gait pattern. Though developed for SCNC use with children, the tripping monitor is directly applicable for other patients, such as adults with Parkinson’s disease or recovering from stroke. The technology also can be extended to other clinical applications of real-world monitoring where proximity is of interest, such as assessing and encouraging coordinated bimanual activity following stroke.

## Data Availability

The raw data supporting the conclusions of this article will be made available by the authors on request. Computer code developed in this work is deposited on GitHub. Access to the GitHub location is restricted, and software (GUI version number 0.1.0, user manual number 0.0.8) will be released on request to Shriners Children’s.

## Abbreviations

The following abbreviations are used in this manuscript:

ADC	Analog-to-Digital Converter
AI	Artificial Intelligence
COMP	Comparator
FSR	Force Sensitive Resistor
GUI	Graphical User Interface
LED	Light-Emitting Diode
MCU	Microcontroller
NFC	Near-Field Communication
PCB	Printed Circuit Board
RFID	Radio Frequency Identification
RSSI	Received Signal Strength Indicator
SCNC	Shriners Children’s Northern California
THE	Tripping Hazard Event
TI	Texas Instruments
TNP	True Negative Percentage
TPP	True Positive Percentage
UID	Unique Identification Number
